# An Unusual Suspect in a Case of Fungal Necrotizing Soft Tissue Infection

**DOI:** 10.7759/cureus.112913

**Published:** 2026-07-18

**Authors:** Erin N Burns, Boris Cehajic

**Affiliations:** 1 Surgery, William Carey University College of Osteopathic Medicine, Hattiesburg, USA; 2 General Surgery, Hattiesburg Clinic, Hattiesburg, USA

**Keywords:** case report, fungal infection, gastrostomy tube, lactobacillus, necrotizing soft tissue infection, pichia, surgical debridement

## Abstract

Necrotizing soft tissue infection (NSTI) is a rapidly progressive and potentially fatal infection that is most commonly bacterial in origin but can also be fungal. We present the case of a 79-year-old woman who developed NSTI after gastrostomy tube (G-tube) manipulation. She presented to the emergency department (ED) with abdominal pain and inflammation, and imaging revealed the G-tube to be positioned within the soft tissues. The patient then underwent emergent surgical debridement while decompensating from septic shock. Tissue cultures identified an unusual combination of *Pichia* and *Lactobacillus* species. The patient improved with repeated debridement and targeted antimicrobial therapy. This case highlights the importance of considering atypical and fungal sources of NSTI, especially in immunocompromised patients, as well as the critical nature of urgent surgical intervention.

## Introduction

Necrotizing soft tissue infection (NSTI) is a rapidly progressive and potentially fatal infection of fascia and subcutaneous tissues. It can present with vague symptoms that pose a diagnostic challenge, but early identification leading to urgent surgical debridement and empiric antibiotic use is the standard of care [1]. The Laboratory Risk Indicator for Necrotizing Fasciitis (LRINEC) is a diagnostic tool that uses C-reactive protein (CRP), white blood cell (WBC) count, hemoglobin (Hgb), sodium, creatinine, and glucose to calculate the probability of a necrotizing soft tissue infection, with a score over 6 having a 92% positive predictive value [2,3,4].

The microbiology that causes NSTI is predominantly bacterial in nature. Type I infections are polymicrobial, usually caused by a combination of aerobic and anaerobic bacteria [5,6]. Type II infections are monomicrobial and most often caused by Group A *Streptococcus *(GAS) [7]. NSTI from a fungal source is also possible but makes up only 1% of cases [8]. *Candida *species are the most common cause of fungal NSTI [9]. Therefore, we present the case of NSTI caused by an unusual fungal and bacterial combination of *Pichia *and *Lactobacillus* species, respectively.

## Case presentation

A 79-year-old woman presented to the emergency department (ED) nine days following gastrostomy tube (G-tube) placement by interventional radiology after failing a swallow study. The day before her current presentation, she had visited the ED to have her G-tube replaced after it had become dislodged. She returned to the ED when the wound surrounding her G-tube became increasingly painful and inflamed after it was repositioned the day prior.

Her relevant medical history included a cerebral stroke eight years prior; type 2 diabetes mellitus; obesity (body mass index 45.71 kg/m²); hypertension; asthma-chronic obstructive pulmonary disease overlap syndrome; and a fungal scalp infection of unknown duration and frequency. She had undergone an anterior cervical discectomy approximately two months prior that had been complicated by possible cranial nerve injury leading to her swallowing difficulties. She had no other abdominal surgeries other than the G-tube placement and a cesarean section. She was a 10-pack-year smoker who had quit seven years ago. 

**Figure 1:**
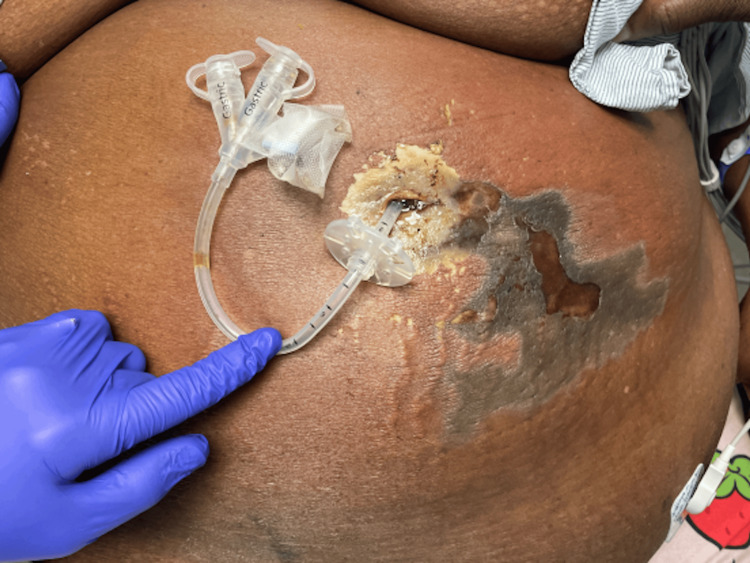
Photograph of the abdominal wound surrounding the gastrostomy tube.

**Figure 2:**
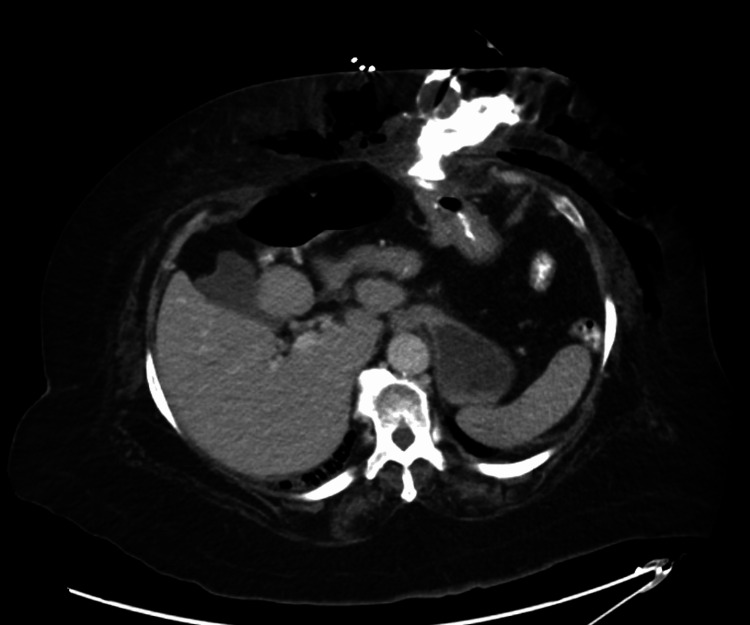
Computed tomography of the abdomen and pelvis with contrast that was taken at admission to the emergency department.

At this current presentation to the ED, she had a temperature of 97.3°F, a blood pressure of 103/44 mmHg, a pulse of 113 beats per minute, and 22 respirations per minute. At this time, her laboratory studies revealed a WBC count of 12.2 × 109/L, Hgb of 11.9 g/dL, blood glucose of 410 mg/dL, blood urea nitrogen (BUN) of 60 mg/dL, creatinine of 2.69 mg/dL, CRP of 494.4 mg/L, procalcitonin of 17.5 ng/mL, and lactic acid of 3.4 mmol/L (Table 1). This calculated to an LRINEC score of 8. Her physical examination revealed a tender abdomen with an erythematous wound surrounding her G-tube site that demonstrated superficial bullae (Figure 1). Computed tomography (CT) of the abdomen and pelvis with contrast (Figure 2) revealed her G-tube to be lying in the soft tissues of her abdomen and extensive soft-tissue emphysema within the left anterolateral abdominal wall, which is concerning for NSTI. At this time, the general surgeon obtained patient consent to proceed with wound debridement in the operating room (OR).

**Table 1:** Relevant laboratory results at time of admission and prior to first debridement.

Parameters	Patient Values	Reference Range
White blood cell	12.2 × 10^9^/L	4.8 – 10.8 × 10^9^/L
Hemoglobin	11.9 g/dL	12.0 – 15.0 g/dL
Blood glucose	410 mg/dL	74 – 106 mg/dL
Blood urea nitrogen	60 mg/dL	7 – 25 mg/dL
Creatinine	2.69 mg/dL	0.60 – 1.30 mg/dL
C-reactive protein	494.4 mg/L	<5.00 mg/L
Procalcitonin	17.5 ng/mL	<0.05 ng/mL
Lactic acid	3.4 mmol/L	0.7 – 1.9 mmol/L

**Figure 3:**
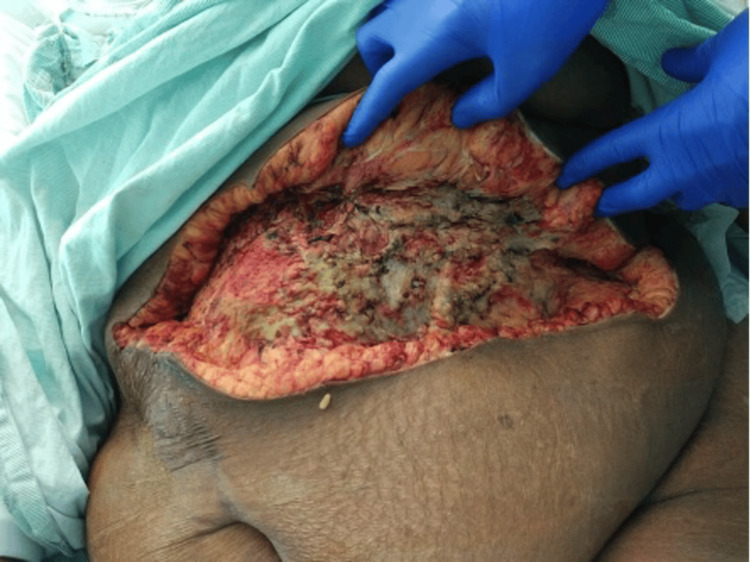
Photograph of the abdominal wound after the first debridement.

The ED physician had begun empiric intravenous vancomycin and Zosyn. Clindamycin and meropenem were added prior to debridement. The patient was then brought to the OR and placed under general anesthesia. Sharp excisional debridement of necrotic skin and subcutaneous tissue down to the fascia was performed until viable tissue was reached. This created a wound that was 49 cm wide x 20 cm long x 5 cm deep (Figure 3). The gastric stoma was sutured closed, and Kerlix soaked in Dakin’s solution was packed inside the wound, and a dry dressing was applied on top of this. The excised tissue was sent to the laboratory for culture. The patient was then brought to the intensive care unit (ICU) while still intubated. The patient required vasopressors at this time. Lactic acid on arrival to the ICU was 5.9 mmol/L (Table 2).

**Table 2:** Lactic acid level at intensive care unit admission following the first debridement procedure.

Parameters	Patient Values	Reference Range
Lactic acid	5.9 mmol/L	0.7 – 1.9 mmol/L

The following morning, approximately 18 hours after the original debridement, the patient was once again brought to the OR under general anesthesia for a dressing change and further debridement of a small amount of remaining necrotic tissue. At this time, the peritoneum and fascia overlying the former gastric stoma were also sutured closed. Fresh Kerlix and dry dressings were reapplied to the wound, and the patient returned to the ICU. Laboratory results that morning revealed a lactic acid of 1.6 mmol/L (Table 3).

**Table 3:** Lactic acid level on postoperative day 1 following the initial debridement.

Parameters	Patient Values	Reference Range
Lactic acid	1.6 mmol/L	0.7 – 1.9 mmol/L

The patient continued to recover in the ICU, eventually being extubated, weaned off pressors, and moved to the med-surg floor on post-operative day 4. Tissue cultures identified *Pichia* and *Lactobacillus* species as the causative organisms of the NSTI using standard clinical laboratory matrix-assisted laser desorption/ionization time-of-flight (MALDI-TOF) mass spectrometry. She was started on a course of Unasyn and micafungin upon the infectious disease physician’s recommendation. No susceptibility testing was performed. Blood cultures were negative.

**Figure 4:**
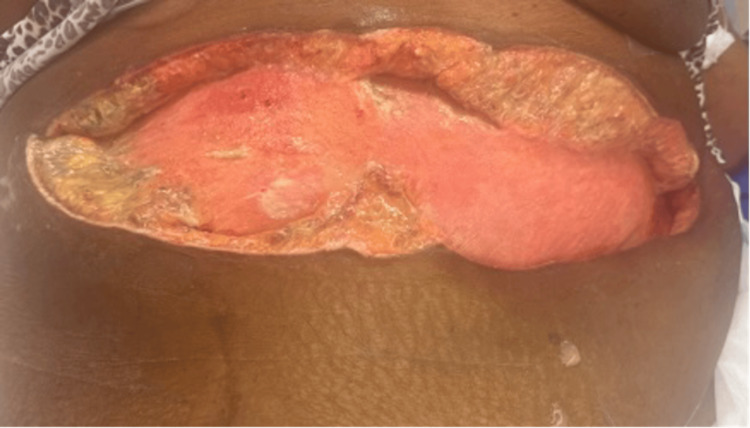
Photograph of the smaller wounds after the third debridement operation.

Six days after the original debridement, the patient was once again brought to the OR, where a few small areas of necrosis were further debrided. Then the wound was partially closed with interrupted nylon sutures that left 2 smaller wounds that measured 17 cm x 12 cm x 3 cm and 30 cm x 14 cm x 3 cm (Figure 4). This time, a wound vacuum was placed. On this day, her laboratory studies revealed a WBC count of 11.1 × 109/L, Hgb of 8.8 g/dL, blood glucose of 109 mg/dL, BUN of 27 mg/dL, and creatinine of 0.94 mg/dL (Table 4).

**Table 4:** Relevant laboratory findings on postoperative day 6.

Parameters	Patient Values	Reference Range
White blood cell	11.1 × 10^9^/L	4.8 – 10.8 × 10^9^/L
Hemoglobin	8.8 g/dL	12.0 – 15.0 g/dL
Blood glucose	109 mg/dL	74 – 106 mg/dL
Blood urea nitrogen	27 mg/dL	7 – 25 mg/dL
Creatinine	0.94 mg/dL	0.60 – 1.30 mg/dL

**Figure 5:**
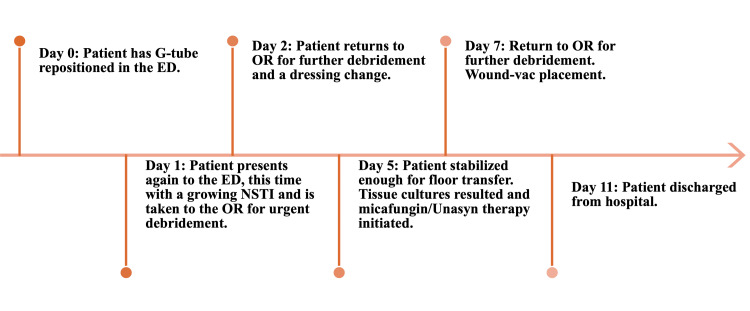
Timeline of hospital admission. G-tube: gastrostomy tube; ED: emergency department; OR: operating room; NSTI: necrotizing soft tissue infection

This wound vacuum remained in place for three days until it began leaking. A new wound vacuum was placed at this time, and the patient was discharged home the next day on continued antimicrobial therapy, Augmentin and micafungin. Home health nurses then took over wound care and wound vacuum changes. The patient has been doing well and has had no readmissions to the hospital at the time of writing. A timeline of inpatient events is summarized in Figure 5.

All laboratory investigations were performed using standard operating procedures and calibrated equipment.

## Discussion

We have presented the case of an elderly patient who suffered from a case of NSTI after G-tube manipulation that resulted in a rapid spread across the majority of the patient’s abdomen. NSTI is recognized as an extremely rare but possible complication of G-tube placement [10]. The patient experienced multiple comorbidities that predisposed her to this complication, such as advanced age, obesity, diabetes mellitus, and the stress of a recent major neck surgery [11, 12].

Because G-tube manipulation was the precipitating event, an enteric source would be suspected. However, one of the two pathogens identified by culture is not. *Lactobacillus* does serve as an important probiotic in the human gut [13] and has been reported to cause NSTI in immunocompromised patients [14]. Conversely, *Pichia* is a yeast that is rarely known to colonize the gastrointestinal tract or otherwise in humans [15]. *Pichia* has been known to cause opportunistic infections in the immunocompromised, with several reports of it causing fungemia in neonatal ICUs [16, 17]. To our knowledge, *Pichia* has not been widely reported as a cause of NSTI.

With fungi being a rare cause of NSTI, it is important not to overlook antifungal coverage when ordering antibiotics for suspected cases of NSTI. Empiric antifungal coverage is not recommended as part of the empiric regimen in immunocompetent patients [18, 19]. Therefore, it is imperative to recognize risk factors for an immunosuppressed state in patients suspicious for NSTI. NSTI of fungal origin has approximately three times the mortality rate compared to that of bacterial origin, and those started on empiric antifungal therapy significantly decreased this risk [9]. 

In this case, targeted antifungal coverage was added after tissue culture results and an infectious disease consult. Early, aggressive surgical debridement contributed to a favorable outcome despite a lack of antimicrobial coverage for *Pichia*.

## Conclusions

We presented a case of NSTI that was precipitated by G-tube manipulation in an elderly patient with diabetes. Cultures identified a rare combination of *Pichia *and *Lactobacillus*. This case underlines the importance of maintaining a high index of suspicion for atypical pathogens in NSTIs, particularly in the setting of a gastrointestinal source. Empiric antifungal coverage may warrant consideration in patients with risk factors for immunosuppression. It also highlights the critical role of early surgical intervention in optimizing outcomes, even with rare pathogens.
